# Time-Dependent Changes of Plasma Concentrations of Angiopoietins, Vascular Endothelial Growth Factor, and Soluble Forms of Their Receptors in Nonsmall Cell Lung Cancer Patients Following Surgical Resection

**DOI:** 10.5402/2012/638352

**Published:** 2012-03-26

**Authors:** Ewa Kopczyńska, Maciej Dancewicz, Janusz Kowalewski, Roman Makarewicz, Hanna Kardymowicz, Agnieszka Kaczmarczyk, Tomasz Tyrakowski

**Affiliations:** ^1^Department of Pathobiochemistry and Clinical Chemistry, College of Medicine, Nicolaus Copernicus University, M. Skłodowska-Curie 9 Street, 85-094 Bydgoszcz, Poland; ^2^Department of Thoracic Surgery and Tumors, Oncology Centre, dr I. Romanowska 2 Street, 85-796 Bydgoszcz, Poland; ^3^Department of Oncology and Brachytherapy, College of Medicine, Nicolaus Copernicus University and Oncology Centre, dr I. Romanowska 2 Street, 85-796 Bydgoszcz, Poland; ^4^Department of Laboratory Diagnostics, Oncology Centre in Bydgoszcz, dr I. Romanowska 2 Street, 85-796 Bydgoszcz, Poland

## Abstract

Even when patients with nonsmall cell lung cancer undergo surgical resection at an early stage, recurrent disease often impairs the clinical outcome. There are numerous causes potentially responsible for a relapse of the disease, one of them being extensive angiogenesis. The balance of at least two systems, VEGF VEGFR and Ang Tie, regulates vessel formation. The aim of this study was to determine the impact of surgery on the plasma levels of the main angiogenic factors during the first month after surgery in nonsmall cell lung cancer patients. The study group consisted of 37 patients with stage I nonsmall cell lung cancer. Plasma concentrations of Ang1, Ang2, sTie2, VEGF, and sVEGF R1 were evaluated by ELISA three times: before surgical resection and on postoperative days 7 and 30. The median of Ang2 and VEGF concentrations increased on postoperative day 7 and decreased on day 30. On the other hand, the concentration of sTie2 decreased on the 7th day after resection and did not change statistically later on. The concentrations of Ang1 and sVEGF R1 did not change after the surgery. Lung cancer resection results in proangiogenic plasma protein changes that may stimulate tumor recurrences and metastases after early resection.

## 1. Introduction

Surgical resection remains the standard treatment for patients with stage I nonsmall cell lung cancer (NSCLC). Even in patients who undergo surgical resection at an early stage of NSCLC, recurrent disease often impairs the clinical outcome. The postoperative 5-year survival rate for stage IA and IB NSCLC was reported to range from 72% to 46% in America and in European countries [1, 2].

The major cause of death among cancer patients is angiogenesis-mediated progression of micrometastases (<1 mm in diameter) to lethal macrometastases (≥1 mm) [3]. Recent findings suggest that the critical regulators of the angiogenic switch are bone-marrow-derived endothelial progenitor cells (EPCs) [3]. The soluble factors (e.g., VEGF) secreted by tumors promote the release of both bone-marrow-derived EPCs (including CD34+ VEGFR2+ cells) and hematopoietic cells (including Tie2 expressing monocytes, CXCR4+VEGFR1+ hemangiocytes) into the peripheral circulation and their recruiting to primary tumor or metastatic lesions [4]. The level of circulating bone marrow-derived EPCs is significantly increased in NSCLC patients and correlated with the clinical situation [5, 6]. EPCs contribute to neovascularization by direct luminal incorporation into sprouting nascent vessels [7] and via paracrine secretion of proangiogenic growth factors [8].

The balance of at least two systems, VEGF-VEGFR and Ang-Tie, regulates vessel formation. VEGF is a potent mitogen for micro- and macrovascular endothelial cells derived from arteries, veins, and lymphatics. It directly induces endothelial cell proliferation, migration, and tube formation. The biological effects of VEGF are mediated by two tyrosine kinase receptors, Flt1 (VEGF R1) and KDR (VEGF R2) [9, 10]. Alternative splicing of these mRNA receptors results in soluble forms of each receptor [11]. The soluble form of VEGF R1 (sVEGF R1) inhibits VEGF activity by sequestering VEGF from signaling receptors and by forming nonsignaling heterodimers with VEGF R2 [12].

Ang1 and Ang2 act through competitive binding to the extracellular domain of the endothelial cell—specific receptor tyrosine kinase Tie2. While Ang1 is an agonist, Ang2 could act as an antagonist or agonist depending on cell type and microenvironmental conditions. Several cell types in various tissues express Ang1. Ang2 is mainly expressed in endothelial cells located at sites of vascular remodeling, and it is stored in intracellular WeibelPalade bodies. Ang2 destabilizes capillary integrity by the disruption of connections between the endothelium and the perivascular cells and alone promotes cell death and vessel regression, but, in conjunction with VEGF, it promotes neovascularization. Ang1 mediates migration, adhesion and survival of endothelial cells—it can elicit an antiapoptotic effect. Generally, it has stabilizing effects on blood vessels and anti-inflammatory properties and acts as a sealing-up factor [13, 14]. According to recent findings (2010) [15], the Ang1/Tie2 signal regulates not only vascular quiescence but also angiogenesis. Downstream signaling of Ang1/Tie2 is dependent on the presence or absence of cell-cell contacts. When cell-cell adhesions are disrupted by VEGF, Ang1 induces the formation of ECM-anchored Tie2 and accelerates angiogenesis cooperatively with VEGF. Soluble Tie2 (sTie2), differently from sVEGF R1, is released from Tie2-expressing ECs by a yet unidentified mechanism. Several factors may influence this shedding process. sTie2 is not capable of signal transduction, but by binding to free Ang1 and Ang2 in the plasma it modulates the impact of these factors on angiogenesis [16].

Clinical studies have demonstrated that surgery is associated with changes in plasma composition that make the patient more susceptible to tumor recurrence [17, 18]. Among plasma proteins whose concentrations are altered after surgery are angiogenic factors.

The aim of this study was to determine the impact of surgery on the plasma levels of the main angiogenic factors during the first month after surgery in non-small cell lung cancer patients. 

## 2. Materials and Methods

### 2.1. Study Population

The study included patients with stage I of non-small cell lung cancer, who underwent tumor resection without any preoperative therapy. These patients were treated in the University Hospital Department of Thoracic Surgery and Tumors, Collegium Medicum in Bydgoszcz, Nicolaus Copernicus University in Toruń, during the years 2008–2010. Three blood samples were taken from each patient: one prior to surgery and others on postoperative days 7 and 30.

The experiment was conducted with the understanding and the consent of the human subject. The study protocol was approved by the Ethical Committee of the Collegium Medicum in Bydgoszcz, Nicolaus Copernicus University in Toruń (Poland).

### 2.2. Blood Sampling and Processing

Ang1, Ang2, sTie2, VEGF, sVEGF R1 concentrations were evaluated in plasma. Four millilitres of blood were taken from elbow vein. EDTA was used as an anticoagulant. Within 30 minutes after the collection, the blood samples were centrifuged at 2–8°C for 15 minutes at 1000 × g. For complete platelet removal, an additional centrifuge at 10,000 × g for 10 minutes was applied. The plasma was stored at −70°C.

### 2.3. Angiogenic Factors Determinations

Ang1, Ang2, sTie2, VEGF, sVEGF R1 concentrations were assayed by commercially available sandwich enzyme-linked immunosorbent assay kits from R&D Systems (Quantikine Human Ang1, Ang2, sTie2, VEGF, sVEGF R1 Immunoassay, R&D Systems Inc., Minneapolis, USA). Kits are designed to measure human Ang1, Ang2, sTie2, VEGF, sVEGF R1 in cell culture supernates, serum, plasma, and other biological fluids. Plasma samples for detection of these five factors were diluted 15-, 5-, 10-, 1-, 10-folded, respectively.

### 2.4. Statistical Analysis

Statistical analysis was done using nonparametric Mann-Whitney's test, Wilcoxon signed-rank test, and Pearson's linear correlation. The results were considered statistically significant for *P* < 0.05.

## 3. Results

The study group consisted of 37 patients with non-small cell lung cancer (10 females and 27 males) aged 42 to 80 (with an average age of 64.7 ± 9.0). By the standards of clinical staging, all patients were classified as stage I (IA-9, IB-28). Twenty-five patients had squamous cell carcinoma, 6-adenocarcinoma and 6-other histological types of tumor (large cell anaplastic and neuroendocrine carcinoma). The most frequent histological grade was G2 (moderately differentiated).

Table 1 presents concentrations of Ang1, Ang2, sTie2, VEGF, and sVEGF R1 before the surgical treatment of lung cancer patients as well as on the 7th and 30th days after tumor resection. The concentration of Ang1 did not significantly change after resection. The median of Ang2 concentration increased on the 7th day (3688.70 versus 2501.30 pg/mL; *P* < 0.0001) and subsequently decreased on the 30th day (2858.25 versus 3688.70 pg/mL; *P* < 0.01), but it was still elevated in comparison to the pretreatment state (2858.25 versus 2501.30 pg/mL; *P* < 0.05). Changes in VEGF concentration were similar to those observed for Ang2; the median of VEGF increased on the 7th day (238.50 versus 124.40 pg/mL; *P* < 0.01). However, the concentration of sTie2 decreased on the 7th day after resection (22,567.0 versus 24,806.0 pg/mL; *P* < 0.05) and did not change statistically later on. The concentration of sVEGF R1 did not change after resection.

In this study, factor correlations were estimated (Table 2). The correlation of Ang1 and Ang2 was weak, both positive and negative, at all observation points (before: *r* = 0.34; after 7: *r* = −0.20; after 30: *r* = 0.15). The correlation between Ang1 and sTie2 was weak and constant (*r* = 0.11; *r* = 0.07; *r* = 0.21); on the other hand, the correlation between Ang2 and sTie2 was repeatedly strong, from slightly negative to highly positive (*r* = −0.04; *r* = 0.32; *r* = 0.63). Correlation coefficients for dependence between Ang1 and VEGF were high (*r* = 0.49; *r* = 0.54; *r* = 0.64); however, for Ang2 and VEGF were from moderately positive to slightly negative (*r* = 0.39; *r* = 0.10; *r* = −0.01). The correlations between VEGF and sVEGF R1 as well sTie2 and sVEGF R1 were moderate.

## 4. Discussion

Many patients with solid tumors who undergo surgical resection develop local recurrences and remote metastases from residual tumor cells postoperatively. There are numerous causes potentially responsible for a relapse of the disease, one of them being extensive angiogenesis.

Although the postoperative angiogenesis response is well established after surgery, the exact causes and sources of angiogenic factors remain unclear. It is proposed that surgery-induced hypoxia, metabolic changes, and inflammation are the three major causes of the production of angiogenic factors in cancer cells as well as in normal cells (e.g., immune cells and fibroblasts) [19]. Upon surgery, hypoxia-inducible factor (HIF) induces the expression of downstream angiogenic factors such as VEGF and Ang2 in residual cancer cells [20]. An HIF-independent angiogenesis pathway is mainly mediated by the transcriptional coactivator PGC-1 *α* (peroxisome-proliferator-activated receptor-gamma coactivator-1-*α*). It is a potent metabolic sensor and regulator induced by a lack of nutrients and oxygen, and it powerfully regulates VEGF expression and angiogenesis in cultured muscle cells and in skeletal muscle in vivo [21]. Surgery-related inflammation is another crucial contributor to angiogenic response following surgical tumor resection. The infiltrating macrophages, immature dendritic cells, and carcinoma-activated fibroblasts are recruited to the surgical site and release numerous angiogenic factors [22].

The aim of this study was to determine the impact of surgery on the plasma levels of the main angiogenic factors during the first month after surgery. VEGF is the most potent promoter of angiogenesis. Ang2 supports VEGF proangiogenic effects. Ang1 stabilizes mature blood vessels. Soluble Tie2 modulates the impact of both proteins on angiogenesis by binding free Ang1 and Ang2 in the bloodstream. VEGFR1 binds VEGF and limits the proangiogenic effects of VEGF at the level of endothelial cells [9–16].

We found that lung cancer surgery is associated with significantly increased plasma VEGF and Ang2 levels postoperatively. VEGF was increased for at least 7 days, Ang2 for 30 days; however, sTie2 was decreased. All of these changes are proangiogenic. Plasma Ang1 and sVEGFR1 concentrations did not change significantly in the postoperative period. Although Ang1 levels did not change and Ang2 increased, the Ang1/Ang2 ratio remained unchanged, but there was a tendency for unfavorable decrease. The Ang1/Ang2 ratio may be more important than angiopoietin levels and it depends on the angiogenic phase; when Ang2 expression is relatively higher than that of Ang1, the induction of tumor angiogenesis is triggered (the “angiogenic switch” is turned on).

Postoperatively increased plasma concentrations of VEGF and Ang2 should be considered in conjunction with those of their inhibitors: sVEGFR1 and sTie2, respectively. The lack of change in sVEGF R1 and a decrease in total sTie2 blood concentration after surgery should result in a greater amount of VEGF and Ang2 free to bind to EC-bound receptors. The VEGF/sVEGFR1 and Ang2/sTie2 ratios were greater both on postoperative days 7 and 30 than the preoperative baseline, and the effective concentrations of VEGF and Ang2 were increased after surgery.

In our study, a correlation between Ang2 and VEGF before surgery was noted (*r* = 0.39). A higher correlation was found between Ang1 and VEGF, both before surgery (*r* = 0.49) and on postoperative days 7 (*r* = 0.54) and 30 (*r* = 0.64).

The problem concerning NSCLC resection has not been addressed in published studies (according to our searches), although Kumara et al. [23–25] investigated this issue in colorectal cancer. We obtained results similar to theirs. As colorectal resection is associated with persistent proangiogenic plasma protein changes (increase of VEGF and Ang2 levels), Shantha Kumara et al. [26] assessed the impact of preoperative and postoperative plasma on in vitro endothelial cell behavior. Postoperative plasma stimulates in vitro endothelial cell growth, migration and invasion, so it may stimulate the growth of residual tumor.

Elevated plasma VEGF levels could have an impact on tumor recurrence, growth of micrometastases, or development of new metastases in the early postoperative period. There are some good reasons to believe that (1) VEGF is a critical cytokine in tumor angiogenesis and a target for therapy [27], (2) tumor growth is intensified by VEGF, which binds to endothelial cells and initiates the process of new blood vessel formation [28], (3) high preoperative blood VEGF levels are associated with more advanced disease and a worse prognosis in lung cancer patients [29, 30].

Similarly to VEGF, an increased plasma Ang2 concentration may contribute to angiogenesis-mediated relapse of the disease due to the following facts: (1) Ang2 destabilizes capillary integrity and, in conjunction with VEGF, promotes neovascularization [31] as well as tumor angiogenesis [32]; (2) increased plasma/serum concentration of Ang2 was observed in various cancers, among others in lung cancer [33], breast and prostate cancer [34], and cervical cancer [35]; (3) in many studies the correlation between Ang2 expression and tumor progression was observed [36–39]; (4) the expression/concentration of Ang2 correlates with various angiogenesis factors, for example, with VEGF in liver cancer [39], which suggests their synergistic role in the regulation of tumor angiogenesis.

## 5. Conclusions

All the presented data seem to suggest that proangiogenic plasma alterations after surgery may result in cancer patients developing recurrent disease either from unrecognized tissue microfoci of tumor cells or from viable cells that persist in the circulation. The inclusion of angiogenic factors in the postoperative diagnostics of lung cancer could lead to improved follow-up therapy and individual antiangiogenic therapy.

## Figures and Tables

**Table 1:** The plasma concentration of Ang1, Ang2, sTie2, VEGF, and sVEGF R1 in controls and lung cancer patients before and after surgical treatment (median, 25–75 percentile).

	Controls	Lung cancer			
		Before	After 7	After 30	*P* value
	Median	Median	Median	Median	
	(25–75 percentile)	(25–75 percentile)	(25–75 percentile)	(25–75 percentile)	
Ang1 [pg/mL]	976.7 (0.0–2094.5)	5682.0 (3118.9–8939.4)	7496.6 (3266.0–11100.0)	5279.1 (990.7–8054.1)	

Ang2 [pg/mL]	1668.3 (1291.4–2139.7)	2501.3 (2138.7–2645.4)	3688.7 (2414.1–4841.70)	2858.2 (2177.2–3502.6)	*P < 0.0001 **P < 0.01 ***P < 0.05

sTie2 [pg/mL]	15788 (11975–30534)	24806 (16545–28939)	22567 (16859–26375)	25 318 (21584–33965)	*P < 0.05

VEGF [pg/mL]	66.1 (38.6–99.4)	124.4 (66.6–251.5)	238.5 (113.3–521.6)	115.5 (42.9–91.0)	*P < 0.01

sVEGF-R1 [pg/mL]	107.8 (86.1–122.9)	95.9 (83.7–120.8)	94.7 (83.0–113.3)	95.9 (88.4–121.6)	

Before: before surgical treatment; After 7: on day 7 after treatment; After 30: on day 30 after treatment.

*Before versus after 7; **after 7 versus after 30; ***before versus after 30.

**Table 2:** The correlation between angiogenic factors before and after surgery (*P* < 0.05).

		Ang1			Ang2			sTie2			VEGF		
		B	A 7	A 30	B	A 7	A 30	B	A 7	A 30	B	A 7	A 30
Ang2	B	*r* = 0.34											
	A 7		*r* = −0.20										
	A 30			*r* = 0.15									

sTie2	B	*r* = 0.11			*r* = −0.04								
	A 7		*r* = 0.07			*r* = 0.32							
	A 30			*r* = 0.20			*r* = 0.63						

VEGF	B	*r* = 0.49			*r* = 0.39			*r* = 0.21					
	A 7		*r* = 0.54			*r* = 0.10			*r* = −0.05				
	A 30			*r* = 0.64			*r* = −0.01			*r* = 0.23			

sVEGF-R1	B	*r* = −0.02			*r* = −0.41			*r* = 0.33			*r* = −0.06		
	A 7		*r* = 0.09			*r* = 0.16			*r* = 0.21			*r* = 0.20	
	A 30			*r* = 0.12			*r* = −0.12			*r* = 0.25			*r* = 0.37

B: before surgery; A 7: on day 7 after surgery; A 30: on day 30 after surgery.

## References

[1] Van Rens MTM, Brutel de la Rivière A, Elbers HRJ, Van den Bosch JMM (2000). Prognostic assessment of 2,361 patients who underwent pulmonary resection for non-small cell lung cancer, stage I, II, and IIIA. *Chest*.

[2] Fang D, Zhang D, Huang G, Zhang R, Wang L, Zhang D (2001). Results of surgical resection of patients with primary lung cancer: a retrospective analysis of 1,905 cases. *Annals of Thoracic Surgery*.

[3] Gao D, Nolan DJ, Mellick AS, Bambino K, McDonnell K, Mittal V (2008). Endothelial progenitor cells control the angiogenic switch in mouse lung metastasis. *Science*.

[4] Gao D, Nolan D, McDonnell K (2009). Bone marrow-derived endothelial progenitor cells contribute to the angiogenic switch in tumor growth and metastatic progression. *Biochimica et Biophysica Acta*.

[5] Nowak K, Rafat N, Belle S (2010). Circulating endothelial progenitor cells are increased in human lung cancer and correlate with stage of disease. *European Journal of Cardio-Thoracic Surgery*.

[6] Dome B, Timar J, Dobos J (2006). Identification and clinical significance of circulating endothelial progenitor cells in human non-small cell lung cancer. *Cancer Research*.

[7] Hilbe W, Dirnhofer S, Oberwasserlechner F (2004). CD133 positive endothelial progenitor cells contribute to the tumour vasculature in non-small cell lung cancer. *Journal of Clinical Pathology*.

[8] Yoon CH, Hur J, Park KW (2005). Synergistic neovascularization by mixed transplantation of early endothelial progenitor cells and late outgrowth endothelial cells: The role of angiogenic cytokines and matrix metalloproteinases. *Circulation*.

[9] Ferrara N (2001). Role of vascular endothelial growth factor in regulation of physiological angiogenesis. *American Journal of Physiology*.

[10] Takahashi H, Shibuya M (2005). The vascular endothelial growth factor (VEGF)/VEGF receptor system and its role under physiological and pathological conditions. *Clinical Science*.

[11] Robinson CJ, Stringer SE (2001). The splice variants of vascular endothelial growth factor (VEGF) and their receptors. *Journal of Cell Science*.

[12] Wu FTH, Stefanini MO, Gabhann FM, Kontos CD, Annex BH, Popel AS (2010). A systems biology perspective on sVEGFR1: its biological function, pathogenic role and therapeutic use. *Journal of Cellular and Molecular Medicine*.

[13] Eklund L, Olsen BR (2006). Tie receptors and their angiopoietin ligands are context-dependent regulators of vascular remodeling. *Experimental Cell Research*.

[14] Brindle NPJ, Saharinen P, Alitalo K (2006). Signaling and functions of angiopoietin-1 in vascular protection. *Circulation Research*.

[15] Fukuhara S, Sako K, Noda K, Zhang J, Minami M, Mochizuki N (2010). Angiopoietin-1/Tie2 receptor signaling in vascular quiescence and angiogenesis. *Histology and Histopathology*.

[16] Reusch P, Barleon B, Weindel K (2001). Identification of a soluble form of the angiopoietin receptor TIE-2 released from endothelial cells and present in human blood. *Angiogenesis*.

[17] Hu Y, Li B, Shi G, Rong C, Gao G (2010). Correlation of postoperative serum VEGF levels with platelet counts in non-small cell lung cancer. *Chinese Journal of Lung Cancer*.

[18] Hu Y, Li B, Song C (2008). Clinical research of perioperative serum VEGF and MMP-9 levels in patients with non-small cell lung cancer. *Chinese Journal of Lung Cancer*.

[19] Kong B, Michalski CW, Friess H, Kleeff J (2010). Surgical procedure as an inducer of tumor angiogenesis. *Experimental Oncology*.

[20] Pouysségur J, Dayan F, Mazure NM (2006). Hypoxia signalling in cancer and approaches to enforce tumour regression. *Nature*.

[21] Arany Z, Foo SY, Ma Y (2008). HIF-independent regulation of VEGF and angiogenesis by the transcriptional coactivator PGC-1*α*. *Nature*.

[22] Carmeliet P (2005). Angiogenesis in life, disease and medicine. *Nature*.

[23] Belizon A, Balik E, Horst P (2008). Persistent elevation of plasma vascular endothelial growth factor levels during the first month after minimally invasive colorectal resection. *Surgical Endoscopy and Other Interventional Techniques*.

[24] Shantha Kumara HMC, Cabot JC, Hoffman A (2009). Minimally invasive colon resection is associated with a transient increase in plasma sVEGFR1 levels and a decrease in sVEGFR2 levels during the early postoperative period. *Surgical Endoscopy and Other Interventional Techniques*.

[25] Shantha Kumara HMC, Grieco MJ, Yan X (2010). Minimally invasive colorectal resection for cancer is associated with a short-lived decrease in soluble Tie-2 receptor levels, which may transiently inhibit VEGF-mediated angiogenesis (via altered blood levels of free Ang-1 and Ang-2). *Surgical Endoscopy and Other Interventional Techniques*.

[26] Shantha Kumara HMC, Feingold D, Kalady M (2009). Colorectal resection is associated with persistent proangiogenic plasma protein changes: Postoperative plasma stimulates in vitro endothelial cell growth, migration, and invasion. *Annals of Surgery*.

[27] Dvorak HF (2002). Vascular permeability factor/vascular endothelial growth factor: A critical cytokine in tumor angiogenesis and a potential target for diagnosis and therapy. *Journal of Clinical Oncology*.

[28] Ito Y, Hasuda H, Terai H, Kitajima T (2005). Culture of human umbilical vein endothelial cells on immobilized vascular endothelial growth factor. *Journal of Biomedical Materials Research Part A*.

[29] Trapé J, Buxó J, De Olaguer JP (2003). Serum concentrations of vascular endothelial growth factor in advanced non-small cell lung cancer. *Clinical Chemistry*.

[30] Shimanuki Y, Takahashi K, Cui R (2005). Role of serum vascular endothelial growth factor in the prediction of angiogenesis and prognosis for non-small cell lung cancer. *Lung*.

[31] Asahara T, Chen D, Takahashi T (1998). Tie2 receptor ligands, angiopoietin-1 and angiopoietin-2, modulate VEGF- induced postnatal neovascularization. *Circulation Research*.

[32] Holash J, Maisonpierre PC, Compton D (1999). Vessel cooption, regression, and growth in tumors mediated by angiopoietins and VEGF. *Science*.

[33] Joo HP, Kwang JP, Young SK (2007). Serum angiopoietin-2 as a clinical marker for lung cancer. *Chest*.

[34] Caine GJ, Blann AD, Stonelake PS, Ryan P, Lip GYH (2003). Plasma angiopoietin-1, angiopoietin-2 and Tie-2 in breast and prostate cancer: A comparison with VEGF and Flt-1. *European Journal of Clinical Investigation*.

[35] Kopczyńska E, Makarewicz R, Biedka M, Kaczmarczyk A, Kardymowicz H, Tyrakowski T (2009). Plasma concentration of angiopoietin-1, angiopoietin-2 and Tie-2 in cervical cancer. *European Journal of Gynaecological Oncology*.

[36] Eggert A, Ikegaki N, Kwiatkowski J, Zhao H, Brodeur GM, Himelstein BP (2000). High-level expression of angiogenic factors is associated with advanced tumor stage in human neuroblastomas. *Clinical Cancer Research*.

[37] Reiss Y, Machein MR, Plate KH (2005). The role of angiopoietins during angiogenesis in gliomas. *Brain Pathology*.

[38] Nakayama T, Hatachi G, Wen CY (2005). Expression and significance of Tie-1 and Tie-2 receptors, and angiopoietins-1, 2 and 4 in colorectal adenocarcinoma: Immunohistochemical analysis and correlation with clinicopathological factors. *World Journal of Gastroenterology*.

[39] Moon WS, Rhyu KH, Kang MJ (2003). Overexpression of VEGF and angiopoietin 2: A key to high vascularity of hepatocellular carcinoma?. *Modern Pathology*.

